# How external stimuli drive physicians’ value co-creation behavior in online health communities: the mediating role of risk-benefit perceptions and moderating role of digital competency

**DOI:** 10.3389/fpubh.2025.1724166

**Published:** 2026-01-06

**Authors:** Yibei Yao, Jindan Cao, Jie Wang, Yujun Zhong, Meiyu Feng

**Affiliations:** School of Public Health, Jilin University, Changchun, China

**Keywords:** digital competency, online health communities, perceived value theory, physicians, value co-creation

## Abstract

**Objective:**

As Online Health Communities (OHCs) become increasingly integral to healthcare delivery, understanding how to actively engage physicians in value co-creation is a critical practical challenge. This study aims to investigate the mechanisms through which external stimuli influence physicians’ value co-creation behavior (VCB) in OHCs. Specifically, it examines the mediating role of risk–benefit perceptions and the moderating role of physicians’ digital competency in this relationship.

**Methods:**

A cross-sectional survey was conducted among physicians in China with experience in OHCs. Based on the SOR framework and perceived value theory, a research model was developed encompassing environmental, technological, patient, and platform stimuli. Data from 547 valid questionnaires were analyzed using partial least squares structural equation modeling to test the hypotheses.

**Results:**

Perceived benefit exerted a positive effect on VCB, whereas perceived risk had a negative impact. With the exception of the non-significant negative effect of patient compliance on perceived risk, all external stimuli significantly influenced these perceptions. Digital competency significantly moderated these relationships, it enhanced the positive effect of perceived benefit on VCB, importantly, buffered the negative effect of perceived risk on VCB.

**Conclusion:**

This study reveals that physicians’ VCB is driven by a nuanced risk–benefit calculus influenced by multilevel external stimuli. Digital competency plays a vital empowering role, helping physicians leverage benefits and mitigate risks. For OHCs operators and policymakers, our findings underscore the necessity of building a trustworthy environment through policy support and security features, while simultaneously implementing targeted training programs to enhance physicians’ digital competency, thereby fostering a vibrant and sustainable online health ecosystem.

## 1 Introduction

The advent of the Web 2.0 era has spurred the flourishing development of Online Health Communities (OHCs), which have become pivotal hubs facilitating the exchange of medical information and social support between healthcare professionals and patients. Physicians, as core contributors of expertise and services, form the bedrock of OHCs’ value proposition. Value co-creation represents a central concept within service science and marketing, transcending the traditional view of value creation as a unilateral output from enterprises. It emphasizes that value is jointly generated through interaction and resource integration between service providers and consumers (1, 2). This conceptual shift signifies a profound transformation in understanding value, moving beyond its inherent attributes in products or services to emphasize “value-in-use” or “contextual value” (3). Within the context of OHCs, a physician’s value co-creation behavior (VCB) refers to activities where they jointly create and enhance the community’s overall value with patients, platform operators, and fellow physicians. This is achieved through sharing expertise, providing consultations, participating in discussions, creating content, and offering feedback on platform issues. Recent advancements in digital health further embed artificial intelligence functionalities within physicians’ workflows, thereby blurring traditional physician-patient boundaries and transforming VCB into a socio-technical collaborative production process. Within OHCs, physicians’ VCB serves not only as an indicator of platform success but also as a crucial manifestation of healthcare service model innovation. Understanding these driving mechanisms holds profound theoretical and practical significance for constructing a vibrant online health ecosystem (4).

Existing research on physician value co-creation within OHCs tends to focus in isolation on individual motivations or platform design factors (5), overlooking the influence of multi-level external stimuli within broader ecosystems. This limits comprehensive understanding of physician’ VCB and the effectiveness of platform design strategies. The ecosystem perspective emphasizes that value creation is no longer confined to a single organization but increasingly requires resource integration and collaboration among diverse actors (6). Within a service ecosystem, multiple actors can coexist and influence value co-creation (7). OHCs are inherently such service ecosystems, encompassing stakeholders including patients, physicians, platform operators, and healthcare institutions (8). At the macro level, the policy environment and social norms constitute significant external stimuli. Digital technology development also provides innovators with novel value creation pathways, expanding existing innovation ecosystem theories (9). At the meso level, platform operators’ economic and social strategies influence physicians’ VCB (10). At the micro level, patients’ interactive behaviors (11) and physicians’ digital competency (12) serve as crucial external stimuli for physicians’ VCB.

To comprehensively understand physicians’ VCB within OHCs, this study employs an ecosystem thinking model incorporating multi-level external stimuli. Our contributions are threefold. First, we developed an integrated model based on the SOR framework to elucidate how multidimensional external stimuli drive physicians’ VCB. Second, we revealed the internal mechanisms of the “organism” black box by establishing the mediating roles of perceived risk and perceived benefits. Finally, we introduced digital competency as a key boundary condition, deepening understanding of “for whom these mechanisms are most effective.”

## 2 Theoretical framework

This study employs the SOR framework as its meta-theoretical foundation, integrating perceptual value theory to construct a multi-level, multi-pathway theoretical integration model. The SOR framework, a foundational theoretical paradigm in environmental psychology proposed by Mehrabian and Russell, provides a systematic explanation for how external environmental stimuli drive specific behavioral responses through individual internal cognitive and affective mechanisms (13). This framework possesses macro-level comprehensiveness and inclusivity, enabling the identification and categorization of multi-source external stimuli, the characterization of individuals’ perception and evaluation processes in response to stimuli, and ultimately the prediction of behavioral outputs.

The origins of perceived value theory can be traced back to the 1980s, when Zeithaml (14) first explicitly proposed the concept of perceived value, defining it as the customer’s overall evaluation of the utility of a product or service based on perceived gains and losses. Within the OHCs context, physicians’ evaluation of VCB constitutes a complex perceptual value calculation. By embedding perceived value theory within the SOR model, this study concretises the “organism” as the physician’s perceived value calculation process. This value judgment determines the individual’s behavior (15).

## 3 Literature review and hypothesis derivation

### 3.1 Environmental dimensions

#### 3.1.1 Humanistic environment

In this study, the human environment refers to the social, professional, and interpersonal atmosphere and influences experienced by physicians when participating in OHCs medical services. According to rational action theory, an individual’s behavioral intentions are influenced by the perceptions of significant others and social normative pressures (16). When physicians perceive positive support and expectations for participation from peers, patients, and healthcare institutions, their own willingness to engage with OHCs often increases accordingly. Based on peer effect theory, colleagues’ active participation provides a demonstration effect, enhancing physicians’ identification with and motivation to engage in OHCs (17). Within this environment of consensus, physicians frequently anticipate that engaging with OHCs will enhance their professional reputation, broaden their academic influence, and yield potential economic returns, thereby amplifying their perception of benefits. Concurrently, group consensus and peer practice help mitigate physicians’ concerns regarding uncertainty risks, such as alleviating apprehensions about professional reputation damage or negative patient feedback. Based on the foregoing analysis, this paper proposes the following hypothesis:

*H1a*: The humanistic environment is positively correlated with physicians' perceived benefits.

*H1b*: The humanistic environment is negatively correlated with physicians' perceived risks.

#### 3.1.2 Policy environment

In this study, the policy environment refers to the national and local-level policy support and regulatory frameworks encountered by physicians when delivering healthcare services through OHCs. Institutional theory posits that regulatory and policy pressures from governments and regulatory bodies constitute key institutional forces shaping organizational and individual behavior (18). A favorable policy environment creates conducive conditions for physician participation in OHCs by providing legitimacy and reducing uncertainty. Numerous countries have introduced relevant policies. For instance, China’s State Council issued the “Guiding Opinions on Promoting the Development of Internet Plus Healthcare,” explicitly supporting medical institutions in collaborating with third-party platforms to deliver internet-based consultations, health advice, and related services. India’s National Digital Health Mission (NDHM) aims to establish a unified national digital health ecosystem, explicitly recognizing and encouraging physicians to provide online services on authorized platforms. These policies not only confer institutional legitimacy upon physicians’ online practice but also generate economic benefits and career development opportunities by expanding practice channels. Simultaneously, they clarify behavioral boundaries and scope of responsibility, thereby alleviating physicians’ concerns regarding legal and compliance risks. Based on the above analysis, this paper proposes the following hypothesis:

*H2a*: The policy environment is positively correlated with physicians' perceived benefits.

*H2b*: The policy environment is negatively correlated with physicians' perceived risks.

### 3.2 Technical dimension

#### 3.2.1 Technical usefulness

According to Holden and Karsh’s definition, perceived usefulness refers to an individual’s subjective evaluation that employing a new technology can enhance their work performance (19). Within the theoretical framework of the Technology acceptance model, perceived usefulness serves as a key antecedent variable influencing user attitudes and behavioral intentions, a relationship extensively supported by empirical evidence in the healthcare sector. Research indicates that perceived usefulness exerts a significant positive influence on users’ trust formation regarding mobile health services (20). Research further demonstrates that this variable effectively predicts healthcare professionals’ technology adoption behavior (21). Another research reveals the causal pathway whereby perceived usefulness enhances physician satisfaction by positively influencing behavioral intention (22). Within the specific context of physicians participating in OHCs, if clinicians perceive the technology as effectively enhancing their work efficiency, optimizing diagnostic and treatment processes, or improving patient care quality, they are more likely to develop higher perceived benefits. This, in turn, strengthens their willingness to use the technology and increases the proactiveness of their participation behavior. On the other hand, technological usefulness also helps reduce physicians’ concerns about uncertainties in the technology’s operational processes and outcomes, alleviating their cognitive burden and anxiety, thereby diminishing perceived risks. Based on the above analysis, this paper proposes the following hypotheses:

*H3a*: Technological usefulness is positively correlated with physicians' perceived benefits.

*H3b*: Technological usefulness is negatively correlated with physicians' perceived risks.

#### 3.2.2 Technical explanatory

Technological explainability in healthcare refers to the process of elucidating the decision-making logic underlying technical models developed from complex medical data. This enables relevant users to comprehend the mechanisms generating conclusions or recommendations, encompassing the data characteristics relied upon, algorithmic principles, and reasoning processes (23). Current research on medical technology explainability primarily unfolds across two dimensions: model explainability and application effectiveness. The former centers on technical interpretability, encompassing model accuracy, stability, and causality, while the latter emphasizes user experience and societal impact, covering safety, fairness, and visualization (24). Given the unique nature of healthcare settings, technologies face stricter requirements regarding transparency, safety, accountability, and fairness (25). Based on the Job requirement control theory, highly interpretable medical technologies can provide healthcare professionals with clear decision-support rationale. This reduces physicians’ uncertainty regarding black-box decision-making, enhances trust in the technology, and alleviates redundant cognitive load, thereby fostering human-machine collaborative innovation (26). Conversely, low interpretability significantly increases perceived risks for physicians, including liability for misdiagnosis, legal disputes, and threats to patient safety (27). Based on the above analysis, this paper proposes the following hypotheses:

H4a: Technological explainability is positively correlated with physicians' perceived benefits.

H4b: Technological explainability is negatively correlated with physicians' perceived risks.

#### 3.2.3 Technical security

Technical security refers to the aggregate of technical and administrative measures implemented by the OHCs platform to protect data from unauthorized access, disclosure, alteration, and destruction. These measures include data encryption, secure authentication, access controls, and compliance audits. According to protection motivation theory, individuals perceive high technical security as generating positive coping intentions (28). Within a secure network environment, physicians can share expertise and engage in medical interactions with greater confidence, thereby effectively amplifying their influence and fostering VCB (29). Conversely, if concerns about technological security exist, physicians perceive significant potential risks extending beyond the technical realm into professional and legal domains, substantially inhibiting their willingness and behavior to participate (30). Based on this, the following hypotheses are proposed:

*H5a*: Technological security positively correlates with physicians' perceived benefits.

*H5b*: Technological security negatively correlates with physicians' perceived risks.

### 3.3 Patient dimension

#### 3.3.1 Patient feedback

In this study, patient feedback refers to patients’ positive, affirming responses—such as evaluations, expressions of gratitude, and recognition—toward physicians’ online services. According to social exchange theory, physicians’ contributions of professional knowledge in OHCs can be viewed as a form of social investment, while patients’ positive feedback constitutes a significant social return. This return primarily manifests as social recognition and enhanced reputation (31), significantly boosting physicians’ perceptions of social (32) and psychological benefits (33), thereby motivating their continued participation in value co-creation. From the perspective of Uncertainty Reduction Theory, the OHCs environment’s lack of contextual cues from offline interactions can lead physicians to perceive uncertainty regarding service effectiveness and acceptance, thereby heightening perceptions of performance and social risk (34). Positive patient feedback, as explicit behavioral signals, effectively reduces such uncertainty and diminishes perceived risk (35). Based on this, the following hypotheses are proposed:

*H6a*: Patient feedback frequency is positively correlated with physicians' perceived benefits.

*H6b*: Patient feedback frequency is negatively correlated with physicians' perceived risks.

#### 3.3.2 Patient compliance

Patient compliance refers to the extent to which patients follow treatment plans, health recommendations, or behavioral guidance provided by physicians (36). Within OHCs, patients’ adoption and implementation of physicians’ online advice not only reflect the quality of physician-patient interactions but also serve as a crucial pathway for physicians to obtain performance feedback and gain experiential insights—key sources shaping physicians’ perceived value (37). When physicians observe patients achieving positive health outcomes due to their recommendations, it significantly enhances their perceived benefits, including professional fulfillment, efficacy, and enhanced professional reputation. High compliance, as a clear positive feedback signal, reduces physicians’ concerns about the unpredictability of treatment outcomes, thereby significantly lowering their perceived risks—such as performance risk, liability risk, and psychological risk (38). Based on this, the following hypotheses are proposed:

*H7a*: Patient compliance is positively correlated with physicians' perceived benefits.

*H7b*: Patient compliance is negatively correlated with physicians' perceived risks.

### 3.4 Platform dimension

#### 3.4.1 Platform usability

The usability of an OHCs platform refers to the extent to which users can easily and efficiently complete required tasks during actual use (39). Research indicates that perceived usability significantly impacts user identification. An easy-to-use platform design substantially reduces the time and effort physicians expend during operations (40). Intuitive interfaces and streamlined workflows enable physicians to quickly review patient records, respond to inquiries, and handle more consultations within the same timeframe, thereby enhancing work efficiency. An easy-to-use platform encourages physicians to participate more actively in knowledge sharing and academic exchanges within the communities (41). Platform usability reduces error rates during physician operations (42). Clear interface layouts and explicit operational guidance minimize mistakes when entering patient information or issuing medical advice, thereby lowering medical risks stemming from operational errors (43). When physicians perceive the platform’s ease of use, they develop greater trust in its overall quality and services. This trust alleviates concerns about platform security and reliability, making physicians more willing to conduct medical activities on the platform. Based on the above analysis, this paper proposes the following hypotheses:

*H8a*: Platform usability is positively correlated with physicians' perceived benefits.

*H8b*: Platform usability is negatively correlated with physicians' perceived risks.

#### 3.4.2 Platform functionality

The platform functionality of OHCs—specifically the completeness and stability of features they offer—plays a pivotal role in fostering value co-creation and influencing user perceptions. Research indicates that platforms promote value co-creation by integrating complementary assets and enhancing readiness, highlighting the critical role of functional configuration (44). Digital business strategies advance value co-creation with multiple stakeholders through functional design (45). Specific platform attributes, such as interactivity and personalization, significantly impact user behavior as external stimuli (46). When platforms offer efficient consultations, convenient publishing, professional communication, and data management capabilities, physicians more readily realize their professional value, thereby enhancing perceived benefits. Conversely, functional deficiencies, operational complexity, or system instability increase usage burdens and frustration, amplifying perceived risks. Based on this analysis, this paper proposes the following hypotheses:

*H9a*: Platform functionality is positively correlated with physicians' perceived benefits.

*H9b*: Platform functionality is negatively correlated with physicians' perceived risks.

### 3.5 Perceived value

Based on perceived value theory, perceived value represents physicians’ comprehensive assessment of benefits and risks when participating in OHCs medical services. This assessment directly influences physicians’ behavioral intentions and actual participation through a benefit–risk weighing mechanism (47). Research notes that physicians actively engage in online healthcare only when perceived benefits outweigh perceived risks (48). This perspective aligns with prior research indicating that individuals perceiving higher professional benefits are more willing to engage actively in their work (49), and that perceived professional benefits significantly predict personal work enthusiasm and satisfaction (50). This phenomenon also resonates with social exchange theory, suggesting that when individuals perceive professional advantages, they are more likely to provide positive feedback and enhance work engagement (51). Perceived risk refers to physicians’ assessment of the uncertainty and severity of consequences associated with VCB in OHCs (52). Specifically, technical failures may lead to flawed medical decisions and increased misdiagnosis risks (53). Online healthcare services involve complex legal issues such as medical liability and patient privacy protection. Physicians may fear legal disputes arising from improper technology use, which could damage their professional reputation (54). This study examines clinical risks, legal risks, and reputational risks—all of which may diminish physicians’ willingness to participate in OHCs. Based on the above analysis, the following hypotheses are proposed:

*H10*: Perceived benefits positively influence physicians' VCB.

*H11*: Perceived risks negatively influence physicians' VCB.

### 3.6 Digital competency

**Figure 1:**
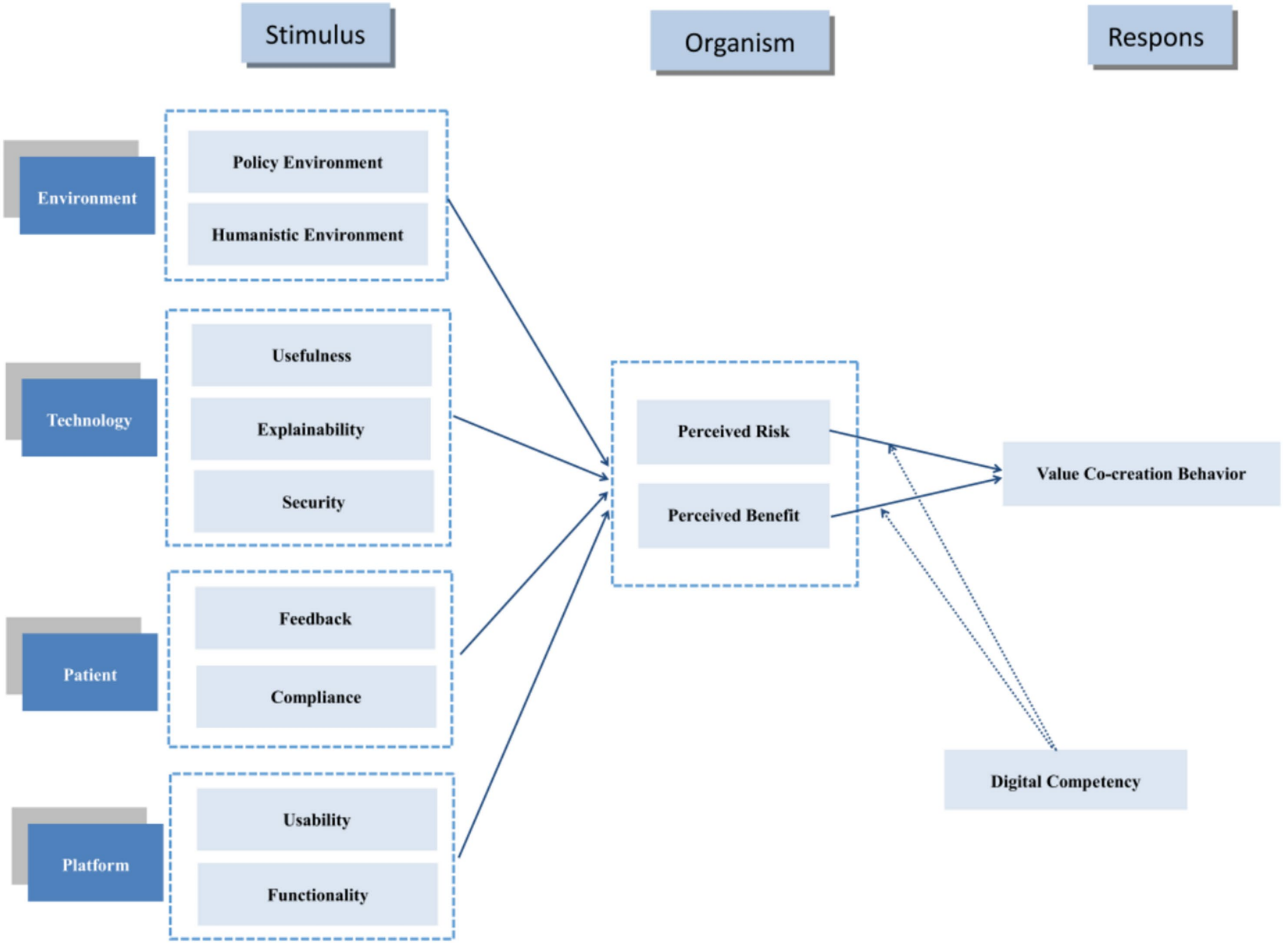
Physicians’ OHCs VCB influence framework.

As a key driver of medical progress, digitalization is fundamentally transforming healthcare, making it increasingly crucial for physicians to understand and evaluate the strengths and limitations of digital tools (55). Physicians’ digital competency refers to the digital-related knowledge, skills, and attitudes required for physicians to integrate digital technologies into the professional setting of patient care (56). Research indicates that physicians’ digital competency influences both self-efficacy and autonomy (57). Physicians with higher digital competency can process patient information more efficiently. This enables them to serve more patients within the same timeframe, thereby increasing work-related income (12). Simultaneously, digital competency helps physicians better leverage online medical education resources for self-improvement, broadening their knowledge base and enhancing diagnostic and treatment capabilities. In turn, improves patient satisfaction and indirectly boosts the physician’s reputation-related income. Physicians with strong digital competency better understand and address potential issues when using digital technologies. Insufficient digital competency among healthcare personnel increases error rates—such as incorrect selections from dropdown menus, reliance on default settings, and overdependence on clinical decision support systems—compromising patient safety while exposing physicians to legal and reputational risks (58). Based on this analysis, this paper proposes hypotheses, with Figure 1 representing the empirical model of this study, and all arrowed paths in the figure represent the theoretical hypothesized relationships proposed in this study.

*H12a*: Physicians' digital competency positively moderates the relationship between perceived benefits and VCB.

*H12b*: Physicians' digital competency negatively moderates the relationship between perceived benefits and VCB.

## 4 Research methodology

### 4.1 Variable measurement

After determining the information required for the questionnaire survey, relevant literature was retrieved, related scales were collected, questions were revised, and they were arranged in sequence to form the initial questionnaire. The questionnaire underwent internal testing and discussion within the research team. Experts from relevant fields such as medical informatics, social medicine, and statistics were invited for pre-test interviews to further refine the questionnaire. Subsequently, a small-sample pre-survey was conducted with 30 research subjects. Existing issues and suggestions were collected through online interviews, face-to-face interviews, and feedback boxes added after the questionnaire. The questionnaire was revised again, and the final version was confirmed. The questionnaire scales primarily drew upon established domestic and international scales or relevant papers, with appropriate modifications made according to the context of this study. All scale items employed a 7-point Likert scale (1 = “Strongly Disagree” to 7 = “Strongly Agree”). Details are shown in Table 1.

**Table 1:** Scale items and rationale.

Dimension		Item	Source
Patient	Compliance	I believe patients will conscientiously follow the health advice or treatment plans provided by doctors on the platform.	Yi and Gong (65)
		I believe patients will complete health management tasks as required by the platform.	
		I believe patients will adhere to the code of conduct and communication guidelines of the online health community.	
	Feedback	I believe patients will provide feedback to doctors regarding the effectiveness or issues encountered during treatment.	
		I believe patients will offer suggestions for improving the platform’s services or functionalities.	
		I believe that when patients receive satisfactory service or assistance, they will offer positive feedback or express their gratitude.	
Platform	Functionality	The platform offers diverse functionalities that facilitate my interactions with patients and other clinicians.	Liu et al. (66)
		The various modules configured on the platform effectively facilitate collaborative communication between myself and users.	
		The platform’s interactive features enable me to co-create value with users.	
	Usability	I find the operational steps and procedures for accessing services through the online health community straightforward and convenient.	Davis (67) Bhattacherjee (68)
		I find it easy to master and become proficient in using the online health community.	
		Overall, I find the operational steps and procedures for accessing services through online health communities straightforward and user-friendly.	
Technology	Utility	I believe the platform’s digital intelligence technology has enhanced my work efficiency.	Venkatesh et al. (69)
		Utilizing the platform’s digital intelligence features has markedly improved my daily clinical efficiency.	
		The AI-assisted diagnosis and precision-targeted recommendations provided by the platform have significantly strengthened my effectiveness in decision-making for complex cases.	
	Explainability	I believe it is currently difficult to provide a clear explanation of the algorithm’s operational mechanisms, making it impossible to fully comprehend the research logic or assess potential risks.	Shin (70) Liu et al. (71)
		I believe that when collecting clinical data, the platform failed to provide timely notification, clear explanation, or deliberately concealed the purpose of data collection, the usage process, and relevant confidentiality measures.	
		I believe that healthcare professionals find it difficult to understand the decision outcomes and treatment plans generated by medical artificial intelligence, thereby failing to ensure the robustness of its clinical application results.	
	Security	The platform’s technology gives me peace of mind when sharing data.	Hwang et al. (72)
		I trust the platform’s technology to securely protect my personal health information.	
		The platform’s technology effectively prevents unauthorized access to my data.	
Environment	Humanistic environment	People around me have advised me to participate in healthcare services within online health communities.	Sun et al. (73) Zhang et al. (74)
		My colleagues engage in healthcare activities within online health communities.	
		The prevailing social climate of health awareness has led me to utilize online health communities to provide services.	
	Policy environment	Regulatory authorities promptly investigate and penalize non-compliant medical practices on the platform, safeguarding my professional security.	Venkatesh et al. (75)
		Current laws and regulations provide clear, actionable guidelines for online consultations, enabling me to participate compliantly.	
		Policies encourage multi-site practice and online follow-up appointments, allowing me to legally expand my service reach and enhance my personal brand.	
Physician	Digital competency	I consider my proficiency in digital technology to be satisfactory.	Golz et al. (76)
		I am able to utilize digital technology to access relevant information.	
		I am able to share information through digital technology.	
		I enjoy using digital technology in my work.	
		I believe digital technology offers significant benefits in enhancing healthcare quality.	
Perceived value	Perceive risk	I believe the absence of physical examinations in online consultations increases the risk of misdiagnosis.	Featherman et al. (77) Kim et al. (47)
		I am concerned that patients may selectively extract and distort my consultation records.	
		I fear negative reviews could damage my professional reputation among peers.	
	Perceived benefits	I consider contributions to online health communities beneficial for my work and studies.	Agrawal et al. (78) Zhang et al. (79)
		I believe participating in online health communities can yield some financial gain.	
		I feel that contributing to online health communities enhances my standing within my professional field.	
Value co-creation behavior	Knowledge contribution	I shall provide professional diagnosis within the online health community.	Peng et al. (80)
		I shall integrate online and offline resources within the online health community to address patient needs.	
		I shall deliver educational medical knowledge within the online health community.	
	Emotional support	I shall manage patients’ emotions within the online health community, alleviating negative sentiments.	
		I shall adapt communication approaches flexibly within the online health community based on individual patient characteristics.	
		I shall adjust communication methods within the online health community according to changes in patients’ medical conditions.	
	Comprehensive patient management	I will deliver consistent, convenient, and timely services within the online health community	
		I will conduct ongoing disease monitoring within the online health community	
		I will guide patients to engage correctly and efficiently in the healthcare process	
	Community citizen	I will share knowledge/experience with colleagues within the online health community	
		I will recommend the community to others based on my positive experience	
		I will provide feedback and suggestions to community administrators	

### 4.2 Data collection and sample

The target group for this study consists of physicians with practical experience providing services in OHCs. To ensure participants possess relevant experience and can offer valuable insights, this study employs a combination of online and offline non-probability sampling methods for participant recruitment. Online recruitment involved distributing questionnaires through professional online communities where physicians congregate, while offline recruitment utilized professional networks and medical conferences. Participation was entirely voluntary, and all participants provided informed consent prior to survey commencement. The sample size determination referenced Kendall’s methodological guidelines, which recommend a minimum sample size of 5 to 10 times the number of variables. The survey questionnaire comprised 50 items related to the construct being measured. Based on this guideline, the target sample size required for robust analysis ranged between 250 and 500 cases. We aimed to exceed the upper limit of this range to enhance the statistical power and stability of the model. The survey was conducted nationwide in China from March to August, 2025, yielding 610 completed questionnaires from medical professionals. To ensure data quality and authenticity, we implemented rigorous data screening procedures. Exclusion criteria included: abnormally short or long completion times; discernible patterns in responses; failure to pass embedded attention check questions. Following this cleaning process, 547 valid physician questionnaires were obtained, yielding an effective response rate of 89.7%.

### 4.3 Data analysis

SPSS 29.0 software was used to analyze demographic characteristics and test for homogeneity of variances. Smart PLS 4.0 software was employed for multicollinearity analysis, reliability and validity testing, and structural equation modeling analysis. This study selected PLS-SEM for several key reasons: First, PLS-SEM maximizes the variance of the dependent variables while ensuring data quality, based on the characteristics of the measurement model. Moreover, its structural assessment properties are not highly restrictive. Therefore, PLS-SEM is well-suited for handling complex research models. The present model incorporates multiple latent variables and includes moderator variables, resulting in a complex structure. Second, PLS-SEM is better suited for theoretical development rather than theoretical testing, aligning with this study’s focus on advancing theory.

## 5 Results

### 5.1 Sample statistics

The demographic information of the 547 questionnaire samples included in the analysis is shown in Table 2.

**Table 2:** Study sample characteristics.

Variable		Frequency	Composition ratio (%)
Gender	Male	312	57
	Female	235	43
Age	Under 30	24	4.4
	31–40	119	21.8
	41–50	232	42.4
	51 and above	162	31.4
Professional title	Resident physician	79	14.4
	Attending physician	156	28.5
	Associate chief physician	211	38.6
	Chief physician	101	18.5

### 5.2 Measurement model evaluation

#### 5.2.1 Common method Bias test

Given that questionnaire data collection relies on self-reported, single-source information, common method variance (CMV) may exist, posing a potential threat to the internal validity of the study. To examine CMV, this study employed Harman’s single-factor test using SPSS 29.0 software, following the methodology proposed by Podsakof et al. (59). Results indicated that unrotated principal component factor analysis yielded 12 factors with eigenvalues exceeding 1 for the physician model. The first factor explained 33.082% of the variance, falling below the 40% critical threshold. This confirms the absence of significant CMV issues.

#### 5.2.2 Multicollinearity analysis

The issue of multicollinearity among variables can influence the results of structural equation modeling. This study employed Smart PLS 4.0 software to examine multicollinearity by calculating the Variance Inflation Factor (VIF) for each variable (60). Results indicate that all variables’ VIF values range from 1.228 to 1.769, falling below the threshold of 10. Therefore, it can be concluded that no severe multicollinearity exists among the variables in this study.

### 5.3 Validity and reliability testing

This study employed SmartPLS 4.0 software to conduct reliability and validity tests on the second-order measurement model. Following the two-stage sequential latent variable score separation procedure (61), the first stage calculated first-order dimension factor loadings, Cronbach’s *α*, composite reliability (CR), and average variance extracted (AVE) to assess internal consistency and convergent validity (62). Table 3 results indicate that Cronbach’s α and CR for all dimensions exceeded the 0.70 threshold, demonstrating good reliability (63). Factor loadings for all observed variables surpassed the acceptable reliability level of 0.6, and AVE values exceeded 0.50, providing sufficient evidence for convergent validity. Subsequently, discriminant validity was systematically examined using the heterogeneity-to-monotonicity (HTMT) ratio (64). The HTMT ratios were significantly below 0.85 (Table 4). Based on these multiple indicators, the discriminant validity of all dimensions is confirmed to be well established.

**Table 3:** Reflective scale accuracy analyzes.

Dimension	First order	Loading	Cronbach’s alpha	Composite reliability (rho_c)	Average variance extracted (AVE)
Knowledge contribution	KC1	0.878	0.849	0.908	0.768
	KC2	0.880			
	KC3	0.891			
Comprehensive patient management	CPM1	0.917	0.925	0.953	0.871
	CPM2	0.901			
	CPM3	0.980			
Digital competency	DC1	0.881	0.929	0.946	0.779
	DC2	0.881			
	DC3	0.884			
	DC4	0.891			
	DC5	0.877			
Emotional support	EC1	0.879	0.899	0.938	0.834
	EC2	0.972			
	EC3	0.886			
Patient feedback	PAF1	0.905	0.885	0.929	0.813
	PAF2	0.899			
	PAF3	0.900			
Platform functionality	PLF1	0.856	0.818	0.892	0.733
	PLF2	0.857			
	PLF3	0.856			
Humanistic environment	HE1	0.882	0.858	0.913	0.778
	HE2	0.892			
	HE3	0.872			
Community citizen	CC1	0.877	0.859	0.914	0.779
	CC2	0.876			
	CC3	0.875			
Perceived benefit	PB1	0.887	0.856	0.912	0.776
	PB2	0.874			
	PB3	0.882			
Policy environment	PE1	0.864	0.837	0.902	0.754
	PE2	0.870			
	PE3	0.870			
Platform usability	PLU1	0.901	0.891	0.932	0.821
	PLU2	0.905			
	PLU3	0.911			
Perceive risk	PR1	0.909	0.893	0.933	0.824
	PR2	0.902			
	PR3	0.912			
Technical security	TS1	0.875	0.86	0.915	0.782
	TS2	0.892			
	TS3	0.885			
Technical utility	TU1	0.841	0.811	0.888	0.725
	TU2	0.860			
	TU3	0.853			
Patient compliance	PAC1	0.827	0.780	0.872	0.694
	PAC2	0.825			
	PAC3	0.847			
Technical explainability	TE1	0.846	0.823	0.894	0.738
	TE2	0.868			
	TE3	0.863			

**Table 4:** Discriminant validity assessment: Heterotrait-Monotrait (HTMT) ratios.

Dimension	CC	CPM	EC	KC	HE	PAF	PLU	PAC	PLF	PB	PR	TS	TU	TE	PE	DC
CC																
CPM	0.664															
EC	0.633	0.567														
KC	0.581	0.513	0.472													
HE	0.467	0.428	0.421	0.484												
PAF	0.544	0.438	0.440	0.497	0.390											
PLU	0.341	0.251	0.301	0.288	0.284	0.200										
PAC	0.488	0.325	0.387	0.412	0.387	0.451	0.389									
PLF	0.456	0.433	0.388	0.484	0.487	0.398	0.331	0.346								
PB	0.566	0.415	0.474	0.547	0.605	0.558	0.512	0.600	0.586							
PR	0.554	0.482	0.467	0.525	0.618	0.623	0.415	0.536	0.578	0.627						
TS	0.501	0.439	0.506	0.520	0.581	0.539	0.334	0.510	0.471	0.658	0.704					
TU	0.545	0.413	0.477	0.509	0.447	0.466	0.320	0.414	0.454	0.586	0.615	0.486				
TE	0.474	0.392	0.412	0.499	0.488	0.540	0.311	0.446	0.423	0.617	0.642	0.574	0.487			
PE	0.534	0.463	0.478	0.597	0.593	0.465	0.360	0.490	0.525	0.681	0.650	0.534	0.478	0.480		
DC	0.275	0.303	0.276	0.239	0.287	0.218	0.188	0.245	0.202	0.204	0.392	0.222	0.233	0.200	0.283	

In the second stage, latent variable scores obtained in the first stage are used as indicators for their corresponding second-order models. The t-value is used to test whether external weights are significantly different from zero. The Variance Inflation Factor (VIF) is employed to examine multicollinearity issues among formative indicators. Generally, a VIF < 5 indicates no severe multicollinearity (64). Based on these results, all dimensions make significant contributions to their constructs, and multicollinearity among first-order dimensions is not severe, suggesting the model estimation is valid and reliable (Table 5).

**Table 5:** Accuracy analysis of formative construct of interactivity.

Formative construct	Item	Outer weight	t-value	VIF
VCB	KC	0.426	7.860	1.428
	CC	0.399	6.054	1.896
	CPM	0.163	2.454	1.733
	EC	0.263	4.389	1.599

### 5.4 Structural model evaluation

#### 5.4.1 Path analysis results

To test the theoretical model and research hypotheses proposed in this study, we employed Smart PLS 4.0 software to calculate the significance levels and confidence intervals of path coefficients. Table 6 details the standardized coefficients, standard errors, t-statistics, *p*-values, LLCI, and ULCI for each path. Analysis reveals that perceived benefits exert a highly significant positive influence on VCB, while perceived risks exert a significant negative influence. All antecedent variables exerted significant positive effects on physicians’ perceived benefits. Among these, the policy environment exerted the largest influence coefficient. Except for patient compliance, whose negative effect on perceived risk failed to pass the significance test, all other antecedent variables exerted the expected significant negative effects on physicians’ perceived risk. Technical security made the most prominent contribution to reducing perceived risk.

**Table 6:** Path analysis results.

Hypothesis	Coefficient	S. D.	t-value	*p*-value	LLCI	ULCI
HE → PB	0.110	0.029	3.737	0.000	0.052	0.168
HE → PR	−0.122	0.033	3.749	0.000	−0.187	−0.057
PLF → PB	0.093	0.033	2.848	0.004	0.028	0.156
PLF → PR	−0.161	0.034	4.685	0.000	−0.227	−0.092
PLU → PB	0.171	0.028	6.054	0.000	0.116	0.226
PLU → PR	−0.083	0.027	3.12	0.002	−0.134	−0.031
PAC → PB	0.131	0.035	3.763	0.000	0.059	0.196
PAC → PR	**−0.059**	**0.041**	**1.436**	**0.151**	**−0.137**	**0.023**
PAF → PB	0.128	0.033	3.825	0.000	0.062	0.195
PAF → PR	−0.120	0.032	3.797	0.000	−0.183	−0.060
PB → VCB	0.397	0.038	10.345	0.000	0.317	0.470
PR → VCB	−0.307	0.042	7.342	0.000	−0.39	−0.226
TS → PB	0.131	0.041	3.191	0.001	0.050	0.211
TS → PR	−0.202	0.037	5.465	0.000	−0.272	−0.128
TU → PB	0.104	0.032	3.201	0.001	0.041	0.167
TU → PR	−0.140	0.030	4.639	0.000	−0.199	−0.081
TE → PB	0.118	0.031	3.776	0.000	0.058	0.181
TE → PR	−0.135	0.037	3.649	0.000	−0.208	−0.065
PE → PB	0.175	0.031	5.586	0.000	0.112	0.237
PE → PR	−0.135	0.029	4.644	0.000	−0.192	−0.079

Boldfaced values represent non-significant results.

#### 5.4.2 Mediation effects results

This study examined the mediating effects of perceived benefits (PB) and perceived risks (PR). Results indicate that, except for the indirect effect of patient compliance on VCB mediated by perceived risks, which was not statistically significant, all other indirect pathways were statistically significant. Among these, the mediating effect of policy environment through perceived benefits was the strongest, while the mediating effect of platform functionality through perceived risks was also notably prominent (Table 7).

**Table 7:** Mediation effects results.

Hypothesis	Coefficient	S.D.	t-value	*p*-value	LLCI	ULCI
TS → PR → VCB	0.062	0.015	4.209	0.000	0.036	0.093
TS → PB → VCB	0.052	0.017	3.023	0.003	0.020	0.087
TU → PR → VCB	0.043	0.011	3.803	0.000	0.023	0.067
TU → PB → VCB	0.041	0.014	3.047	0.002	0.016	0.069
TE → PR → VCB	0.041	0.012	3.324	0.001	0.019	0.068
TE → PB → VCB	0.047	0.013	3.527	0.000	0.022	0.074
PE → PR → VCB	0.041	0.011	3.719	0.000	0.022	0.065
PE → PB → VCB	0.069	0.014	4.973	0.000	0.043	0.098
HE → PR → VCB	0.038	0.012	3.160	0.002	0.016	0.062
HE → PB → VCB	0.044	0.013	3.451	0.001	0.020	0.070
PLF → PR → VCB	0.050	0.012	4.088	0.000	0.027	0.074
PLF → PB → VCB	0.037	0.013	2.752	0.006	0.011	0.064
PLU → PR → VCB	0.025	0.010	2.675	0.007	0.008	0.046
PLU → PB → VCB	0.068	0.014	4.974	0.000	0.042	0.095
PAC → PR → VCB	**0.018**	**0.013**	**1.383**	**0.167**	**−0.007**	**0.045**
PAC → PB → VCB	0.052	0.015	3.426	0.001	0.022	0.081
PAF → PR → VCB	0.037	0.011	3.346	0.001	0.017	0.061
PAF → PB → VCB	0.051	0.015	3.440	0.001	0.023	0.081

Boldfaced values represent non-significant results.

#### 5.4.3 Moderation effect results

**Figure 2:**
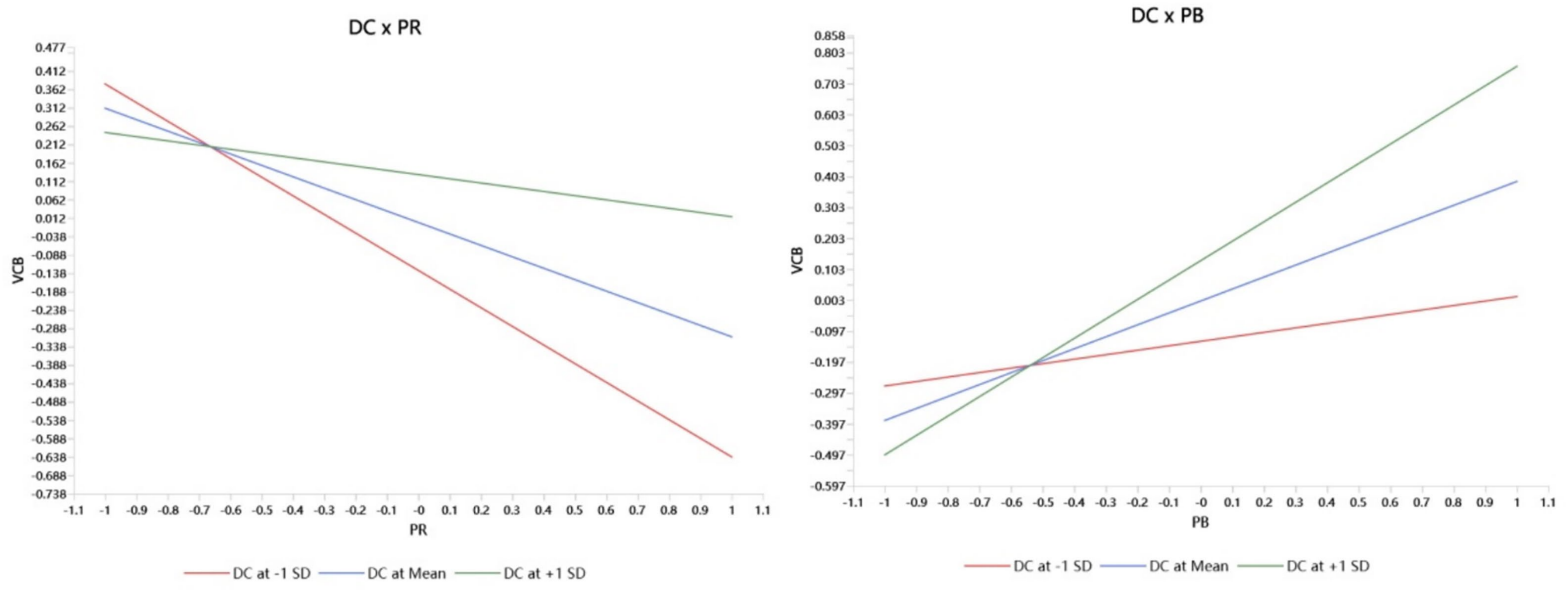
Moderating effects of digital competency.

This study conducted an in-depth analysis of the moderating effect of digital competency on the relationship between perceived benefits, perceived risks, and VCB. Results indicate that digital competency significantly moderates both pathways. First, digital competency substantially enhances the positive influence of perceived benefits on VCB. Simple slope analysis reveals that for physicians with higher digital competency (+1 SD), perceived benefits exert the strongest positive impact on VCB; conversely, this positive effect is significantly weakened for physicians with lower digital competency (−1 SD). Second, digital competency significantly mitigated the negative impact of perceived risk on VCB. Simple slope analysis indicates that when physicians possess higher digital competency, the inhibitory effect of perceived risk on VCB is markedly reduced. Conversely, for physicians with lower digital competency, the negative impact of perceived risk is most pronounced (Figure 2).

## 6 Discussion

This study constructs a multi-level, multi-pathway integrated model based on the SOR framework and Perceived Value Theory, systematically empirically examining the formation mechanism of VCB among physicians in OHCs.

First, the study found that perceived benefits and perceived risks are the most critical drivers of physicians’ VCB, with their mechanisms aligning with the fundamental assumptions of perceived value theory. Physicians’ participation in value co-creation essentially involves a precise “benefit–risk” calculation. Positive participation is more likely to occur when perceived professional, emotional, and social benefits outweigh perceived clinical, legal, and reputational risks.

Second, all multi-level external stimuli—except for the negative influence of patient compliance on perceived risk, which failed to pass significance testing—were confirmed to effectively influence VCB indirectly by enhancing perceived benefits and reducing perceived risks. This fully validates the SOR framework’s effectiveness and inclusiveness in explaining user behavior within complex digital health environments. Notably, the policy environment most significantly enhances perceived benefits, while technical security most substantially reduces perceived risks. This outcome underscores the foundational importance of providing clear institutional legitimacy endorsements at the macro level and constructing robust security barriers at the technical level for stimulating physician participation.

An intriguing finding is that patient compliance failed to significantly reduce physicians’ perceived risks (H7b not supported). This may stem from the asynchronous interaction nature of OHCs, where temporal and spatial separation exists between patients’ online compliance behaviors and offline health outcomes. This prevents physicians from obtaining clear, immediate feedback on the ultimate effectiveness of their recommendations, thereby failing to effectively alleviate their deep-seated concerns about performance risks and outcome uncertainties. This finding suggests that in digital healthcare settings, the traditional concept of compliance may require reevaluation. Platform design may need to incorporate more effective closed-loop feedback mechanisms to bridge the verification gap between online behaviors and offline outcomes.

In assessing VCB as a formative construct, the external weights of its dimensions varied. Knowledge contribution and community citizenship demonstrated the highest contributions, indicating that within the OHCs context, physicians perceive sharing professional knowledge and actively participating in community building as the core manifestations of value co-creation. In contrast, the weight for comprehensive patient management was relatively lower. A plausible explanation is that achieving comprehensive and continuous patient management in asynchronous, non-continuous online interactions is more challenging compared to offline settings, thus diminishing its relative importance in physicians’ overall perception of VCB. Emotional support played a stable and significant role. These differences in weights substantiate the nature of VCB as a formative construct—its dimensions collectively define, rather than are equivalent to, the behavior, and their relative importance can vary across contexts.

The most significant theoretical finding lies in the moderating role of digital competency. This study confirms that digital competency not only directly promotes VCB but also plays a crucial “enabling” moderating role. Physicians with high digital competency are better equipped to translate perceived benefits into tangible actions. They also demonstrate greater proficiency in leveraging their digital skills to mitigate and circumvent potential risks, significantly reducing the negative impact of risk perception. This indicates that in the digital health era, physicians’ technical capabilities have become a critical boundary condition regulating the relationship between their psychological cognition and external behavior. Physicians with high digital competency can extract greater value from identical platform environments.

## 7 Theoretical significance and practical significance

### 7.1 Theoretical significance

(1) Deepened and expanded the application of the SOR framework in the digital health domain. This study successfully integrates the classic SOR framework with perceived value theory. It not only identifies and categorizes the multi-source, multi-level external stimuli influencing physician value co-creation but, more critically, unlocks the organism black box. Specifically, it reveals the dual-path parallel mediation mechanism of perceived benefits-perceived risks, providing a more refined theoretical lens for understanding the complex decision-making processes of professional users in digital environments.
(2) Proposed a novel perspective of digital competency as an enabling moderator. This study breaks away from the traditional research paradigm that views digital competency as a mere antecedent or direct driver. Empirical evidence demonstrates that digital competency, as a crucial individual capability, can amplify positive cognition and buffer the impact of negative cognition on behavior.
(3) Achieved a systematic integration of drivers for physicians’ VCB. This study incorporates multidimensional factors, including policy, humanities, technology, patients, and platforms, into a unified theoretical model for testing. This approach avoids the fragmented limitations of prior research, providing a comprehensive and verifiable theoretical foundation for future studies on value co-creation within OHCs.

### 7.2 Practical significance

(1) Implications for platform operators and designers: Establish a dual-driven trust system combining policy and technology. Platforms should actively collaborate with medical institutions and industry associations to translate national policies into clear operational guidelines and standardized templates embedded within workflows, thereby enhancing physicians’ sense of institutional security. Concurrently, sustained investment in resources is essential to improve the platform’s technical security and transparency, fundamentally reducing physicians’ risk perception. Given the critical moderating role of digital competency, platforms should establish a digital competency diagnostic system for physicians. For those with low digital competency, provide “lightweight” interfaces, operational guidance, and specialized skill training to lower usage barriers. Simultaneously, develop advanced features and value creation opportunities for highly digitally literate groups to maximize their perceived benefits.
(2) Implications for policymakers and healthcare institutions: Relevant government departments should consistently issue and clarify detailed regulations supporting the development of internet-based healthcare. Through official channels, they should widely publicize these policies to provide strong legitimacy backing for physicians’ online practice, eliminating policy uncertainty. Healthcare institutions should incorporate digital health competency as a key component of physicians’ professional competencies into their assessment and training systems. They should encourage and support physicians in enhancing their digital technology application capabilities to better adapt to and lead the digital healthcare transformation.
(3) Implications for Physicians: This study provides directional guidance for physicians engaged in VCB within OHCs. Physicians are advised to proactively enhance their digital competency through specialized training and practical experience. Concurrently, they should actively monitor and understand relevant policies and regulations, exploring diverse value co-creation models within compliance frameworks to build and consolidate sustainable personal online professional brands and influence.

## 8 Limitations

The data for this study were collected from physicians in China. The cultural background of the sample and the characteristics of the healthcare system may limit the generalizability of the findings. Future research could conduct cross-cultural comparisons across different countries and healthcare systems to test the robustness of the model. Furthermore, the use of cross-sectional data does not allow for strict inference of causal relationships between variables. Longitudinal studies or experimental methods could be employed in future research to further validate causal chains. This study primarily focused on physicians’ subjective perceptions and behavioral intentions. Future research could incorporate objective behavioral data for more comprehensive measurement. Finally, this study analyzes the physician group as a relatively homogeneous entity. However, different clinical specialties and the types of diseases they primarily manage may profoundly influence physicians’ value co-creation behavior patterns within online health communities. Future research could delve deeper into the role these characteristics play in physicians’ value co-creation behaviors.

## Data Availability

The raw data supporting the conclusions of this article will be made available by the authors, without undue reservation.
